# An ultraviolet-C (UV-C) light device is effective for low-level disinfection of surgical site marking pens with UV-C transmissible caps

**DOI:** 10.1017/ash.2025.13

**Published:** 2025-02-10

**Authors:** Amelia L. Milner, Jennifer L. Cadnum, Jennifer M. Hailes, Curtis J. Donskey

**Affiliations:** 1 School of Public Health, University of Pittsburgh, Pittsburgh, PA, USA; 2 Research Service, Louis Stokes Cleveland VA Medical Center, Cleveland, OH, USA; 3 Geriatric Research, Education, and Clinical Center, Louis Stokes Cleveland VA Medical Center, Cleveland, OH, USA; 4 Department of Medicine, Case Western Reserve University School of Medicine, Cleveland, OH, USA

## Abstract

Single-use plastic pens are commonly used to mark surgical sites on the skin of patients. In laboratory testing, an ultraviolet-C (UV-C) light device was effective for decontamination of marking pens with plastic caps designed to allow transmission of UV-C. Decontamination of marking pens could reduce plastic and carbon footprints.

## Introduction

The healthcare system contributes substantially to greenhouse gas emissions in the United States.^1^ Plastics account for 30% of healthcare waste, and many plastic items are single-use disposables.^2^ In addition to greenhouse gas emissions, plastic waste causes adverse environmental effects.^2,3^ Thus, there is increasing interest in approaches that might allow reuse of plastic items in healthcare.^4^

Single-use plastic pens are used to mark surgical sites on skin. These pens are classified as non-critical items requiring only low-level disinfection because they only contact intact skin.^5^ Low-level disinfection of these pens is challenging because liquid disinfectants might damage the tip of the pen and technologies such as ultraviolet-C (UV-C) light do not penetrate the plastic cap that covers the pen tip. Moreover, because marking pens are relatively inexpensive, a disinfection process would have to be inexpensive and easy to use to be cost-effective. Here, we tested the efficacy of a UV-C light device for low-level disinfection of marking pens with novel plastic caps designed to allow transmission of UV-C light to the pen tip.

## Methods

### Description of the test device

The Steri-Write system (Steri-Write, North Canton, OH) is a small portable device designed for semi-automated pen decontamination using 265-nm UV-C.^6^ Pens placed in the top of the device are automatically loaded onto a conveyer system that rotates the pens to provide UV-C exposure to all sides. The total exposure time is adjustable. After decontamination, pens are dispensed at the base. In a previous study, the device reduced bacteria and viruses inoculated on writing pens.^6^ The manufacturer’s suggested retail price for the device is less than $1,000 and the estimated lamp life is more than 5 years.

For the current study, we used marking pens with caps made from a proprietary plastic that allows transmission of ∼70% of UV-C light, providing decontamination to the area under the cap including the felt tip of the marker. The suggested retail price for the pen is $2.00. Based on testing completed by the manufacturer, it is estimated that each marker would provide 400 or more uses before needing replacement due to loss of ink; no degradation of the plastic occurs with this amount of exposure.

### Evaluation of efficacy in reducing pathogens

We tested the efficacy of the device against a clinical methicillin-resistant *Staphylococcus aureus* (MRSA) isolate and *Escherichia coli* (American Type Culture Collection number 15597) using a modification of the American Society for Testing and Materials standard quantitative carrier disk test method (ASTM E-2197-02).^7^ Ten μL aliquots containing ∼10^6^ log_10_ colony-forming units (CFU) of the test organisms in phosphate-buffered saline with 5% fetal calf serum were inoculated onto the felt writing tip or the outside body of the pen; for the pen tip, the pen was capped after inoculation. After air drying for 30 minutes, the pens were exposed to UV-C cycles of 45, 180, or 360 seconds. The body of the pen was sampled with pre-moistened cotton-tipped swabs that were vortexed for 1 minute in 200 µL of phosphate-buffered saline with 0.02% Tween. Serial dilutions were plated on selective media including CHROMagar *Staph aureus* with 6 μg/mL cefoxitin for MRSA and MacConkey agar for *E. coli*. The pen tip was sampled by direct imprint onto selective media. Experiments were completed in triplicate with 3 control and 3 experimental pens for each test organism. Log_10_ CFU reductions were calculated in comparison to untreated controls. A reduction of ≥3 log_10_ in comparison to untreated controls was considered effective.^8^

### Estimation of the carbon and plastic footprints of the markers

We estimated the cost of the markers and the carbon and plastic footprints for our hospital. Information on the number and cost of markers purchased each year was obtained from hospital purchasing records. The plastic footprint was calculated as the total amount of plastic waste discarded per year. The carbon footprint was approximated using the conversion rate of 1 kg of plastic to 5 kg of CO_2_.^9^

## Results

**Figure 1.**
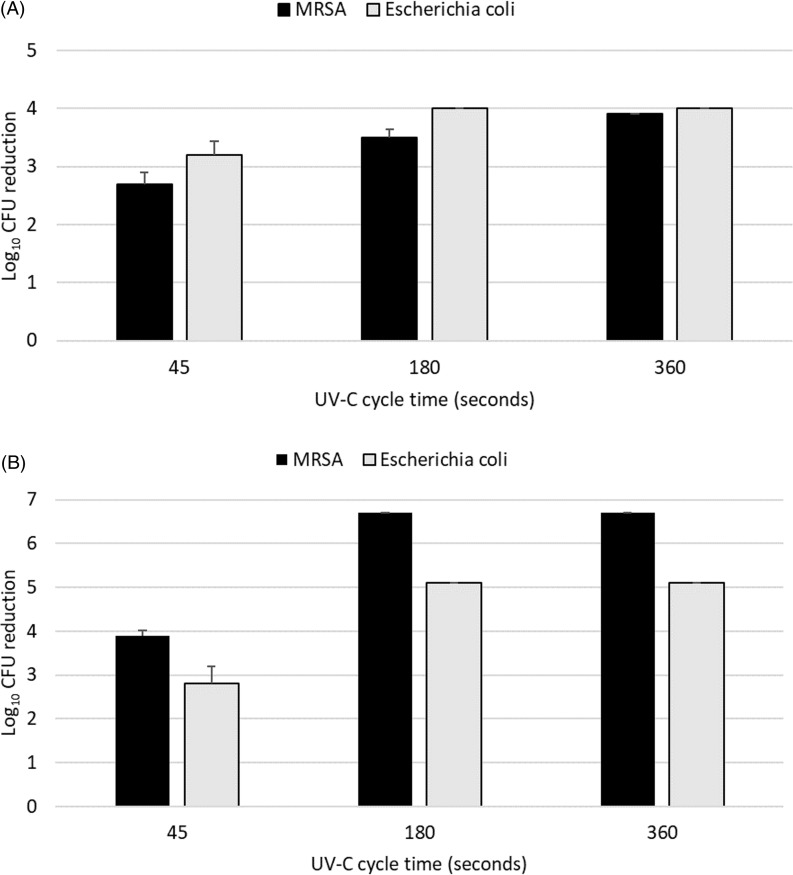
Efficacy of 45-, 180-, and 360-second ultraviolet-C (UV-C) light cycles in reducing pathogens inoculated on the felt writing tip (A) and body (B) of surgical site marking pens. CFU, colony-forming unit. Error bars show standard error.

Figure 1 shows the efficacy of the 45-, 180- and 360-second cycles in reducing MRSA and *E. coli* on inoculated pens. The 180- and 360-second cycles consistently reduced MRSA and *E. coli* by >3 log_10_ CFU, whereas the 45-second cycle did not. The 180-second cycle reduced the test organisms to undetectable levels in all experiments except for MRSA on the pen tip; no organisms were recovered in any experiment with the 360-second cycle.

Our 215-bed hospital orders ∼18,000 marking pens (Surgical Skin Markers) each year- with an annual cost of ∼$19,484 ($1.08 per marker). The individual markers weigh 5.7 grams resulting in a plastic footprint of approximately 102.4 kg of plastic each year. Based on the plastic-to-CO_2_ conversion, approximately 511.2 kg of CO_2_ are released yearly due to use and disposal of single-use surgical markers at our facility. Based on the manufacturer’s suggested pricing ($2.00 per pen) and estimate of 400 uses per pen, use of the technology would require 45 marking pens each year at a cost of $90 with a plastic footprint of 0.26 kg.

## Discussion

Single-use plastic items contribute substantially to the plastic and carbon footprints of healthcare facilities.^1–3^ We demonstrated that a UV-C light device was effective for low-level disinfection of marking pens with plastic caps that were modified to allow transmission of UV-C. A 360-second cycle consistently reduced the test pathogens to undetectable levels. These findings suggest that use of the UV-C device in combination with the modified plastic caps could allow multiple reuses of marking pens, thereby reducing costs as well as carbon and plastic footprints.

The use of plastic that allows transmission of UV-C light is a novel approach to enhance the disinfection capability of UV-C. UV-C is ineffective for disinfection of the internal components of many devices with lumens due to lack of penetration through plastic. Additional studies are needed to identify other potential applications of the UV-C transmissible plastic for disinfection of non-critical or semi-critical items.

Our study has some limitations. We only tested efficacy against MRSA and *E. coli*. UV-C light is less effective against *Candida auris* and *Clostridioides difficile* spores.^6,8^ However, in settings where such organisms are a concern, the device could be adjusted to provide a longer UV-C cycle. We did not calculate the time required for use of the device in a real-world setting. However, we anticipate that the time requirement would be minimal if the device were placed in the area where marking pens are used. We did not culture markers that had been used to assess the level of contamination in real-world settings. It is plausible that contamination of the marker tip may be uncommon given that the ink component of many commercial markers contains gentian violet with anti-bacterial and anti-fungal properties.^10^ Finally, the UV-C transmissible cap is currently only available for pens used with the UV-C light device. However, in the future the same type of plastic could be used to make caps for other marking pens.

In summary, the UV-C light device was effective for low-level disinfection of marking pens. Use of the UV-C device could potentially reduce the carbon and plastic footprints associated with these pens. Additional studies are needed to investigate the potential for UV-C transmissible plastics to facilitate the use of UV-C for disinfection of other plastic items used in healthcare.
